# Variations in the structural and functional properties of flaxseed gum from six different flaxseed cultivars

**DOI:** 10.1002/fsn3.2566

**Published:** 2021-09-16

**Authors:** Xuejiao Ren, Huili He, Tuoping Li

**Affiliations:** ^1^ College of Food Shenyang Agricultural University Shenyang China; ^2^ College of Food Science and Technology Jinzhou Medical University Jinzhou China

**Keywords:** flaxseed cultivar, flaxseed gum, Fourier‐transform infrared spectroscopy, scanning electron microscopy, X‐ray diffraction, zeta potential

## Abstract

Although flaxseed gum (FG) has been widely studied, the differences in its structure and function with respect to various flaxseed cultivars remain unclear. In this study, our objective was to examine the differences between FG samples obtained from different flaxseed cultivars based on their structural and functional properties. Specifically, FG samples from the different cultivars were extracted via hot water extraction followed by ethanol precipitation. Thereafter, they were analyzed via zeta potential measurements, Fourier‐transform infrared (FTIR) spectroscopy, X‐ray diffractometry (XRD), and scanning electron microscopy (SEM). The results demonstrated that the different cultivars showed significantly different FG yields (*p* < .05; range, 5.83%–7.36%). Further, the FTIR spectra of the FG samples were slightly different but showed typical polysaccharide absorption peaks. Furthermore, the XRD patterns obtained predominantly showed an amorphous region and a small crystalline region, while the SEM images obtained at 1,000× magnification revealed that the samples had smooth and irregular surfaces, with a scaly structure. However, at 20,000× magnification, the FG samples showed slight structural differences. Additionally, the zeta potentials of the FG samples (range, −19.4 to −30.6 mV; *p* < .05) were cultivar‐dependent and indicated the presence of negatively charged macromolecules. This implies that the FG samples from the different cultivars show diverse structural properties. Our findings not only provide useful information regarding FG samples extracted from different cultivars but also serve as a theoretical basis for the application of FG in food processing.

## Graphical abstract

Flaxseed gum samples from the different cultivars were extracted via hot water extraction followed by ethanol precipitation. Thereafter, they were analyzed via zeta potential measurements, Fourier‐transform infrared (FT‐IR) spectroscopy, X‐ray diffractometry (XRD), and scanning electron microscopy (SEM).

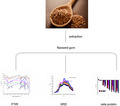

## 1 INTRODUCTION

Flaxseed gum (FG), which is predominantly present in the outermost layer of the seed, accounts for approximately 9% of the dry base weight of the seed, depending on the flaxseed variety and the planting area (Kaushik et al., 2017). However, the composition of the FG depends on the variety, production practice, environmental factors, and storage conditions (Liu et al., 2016). Further, it has also been observed that the purity and yield of FG are closely related to the method by which it is extracted, as well as the amount of target compound that can be recovered from it without degradation (Zhang et al., 2016). FG is primarily concentrated in flaxseed shell, which are composed of dietary fibers and a small amount of digestible sugar. Furthermore, FG is generally difficult to digest, given that it contains both soluble and insoluble fibers, which comprise 7%–10% of the seed's nutrient composition and 25% of its total carbohydrate content. Because FG consists of plant polysaccharides, the various components adhere to each other in the presence of water (Dubois et al., 2020). As a hydrophilic colloid, it has certain functional properties that allow it to be used as a thickening agent in food processing and as a gelatinizer when added to food. Additionally, its physiological activities can enhance weightloss (Luo et al., 2018) and lower cholesterol levels (Kristensen et al., 2012). Numerous studies with a focus on the characterization and analysis of FG have been conducted (Ding et al., 2018). It has also been reported that the extraction conditions of FG significantly affect its composition, particularly its monosaccharide and protein contents. For example, lower extraction temperatures (30–50℃) are associated with an increase in the neutral polysaccharide content of the FG sample, whereas higher extraction temperatures (70–90℃) are associated with an increase in its acidic polysaccharide and denatured protein contents; these compositional changes affect its functional characteristics (Kaushik et al., 2017). Reportedly, high‐temperature extraction can result in an increase in the yield of FG, the inactivation of microorganisms and enzymes, protein denaturation, and a decrease in the production of other compounds. Conversely, low‐temperature extraction can reduce energy costs and avoid microbial inactivation and protein denaturation.

The effects of different genes on the chemical composition of FG have also been investigated. Liu et al. (2016) analyzed six varieties of flaxseed and showed that FG yield, as well as FG neutral and acidic polysaccharide contents varied in the ranges 9.33–13.62 g/100 g, 367–592 mg/g, and 89–181 mg/g, respectively. Further, Cakmak et al. reported that FG extracted from flaxseed shell contains numerous high molecular weight heteropolysaccharides (Cakmak et al., 2021). Additionally, the monosaccharides in FG primarily include xylose, rhamnose, glucose, galactose, arabinose, and fucose, with their compositions and contents varying from one flaxseed cultivar to another. Furthermore, the xylose to rhamnose ratio of FG can reflect the proportion of acidic and neutral polysaccharides it contains (Devi & Bhatia, 2019). Cui et al., (1994) and Bernacchia et al., (2014) compared the chemical composition of FG samples from different sources and reported that the neutral sugar content of golden flaxseed is higher than that of brown flaxseed, while its acidic sugar content (e.g., rhamnose and galacturonic acid) is lower than that of brown flaxseed.

As a water‐soluble colloid, FG has better foam stability than colloidal substances, such as gum arabic and xanthan gum (Wang et al., 2010). Studies have also demonstrated that at the same concentration, an FG solution with a high neutral polysaccharide content exhibits shear thinning and weak gel characteristics, while that of FG with a high acidic polysaccharide content shows typical Newtonian fluid behavior (Elboutachfaiti et al., 2017; Qian et al., 2012). It has also been observed that the composition of monosaccharides and the structure of polysaccharides are the main factors that affect the rheology and emulsification of linseed gum.

This study was conducted to extract, isolate, characterize, and explore the structural properties of FG from different flaxseed cultivars. The FG yields corresponding to the different cultivars were also determined. The study provides fundamental information on the structural properties of FG as well as evidence to support its application in food processing. Additionally, the knowledge on cultivar‐dependent FG characteristics here presented can facilitate the identification of high‐quality flaxseed varieties for specific applications as well as the selection of more suitable FG varieties for food processing.

## 2 MATERIALS AND METHODS

### 2.1 Materials

Six flaxseed *(Linum usitatissimum L.)* cultivars (Huamin flaxseed from Nan'an, Fujian [FG1], organic flaxseed from Chaoyang, Liaoning [FG2], gold flaxseed from Wulanchabu, Inner Mongolia [FG3], brown flaxseed from Wulanchabu, Inner Mongolia [FG4], gold flaxseed from Jinchang, Gansu [FG5], and brown flaxseed from Jinchang, Gansu [FG6]) were investigated in this study. After harvesting, their seeds were placed in sealed bags in a desiccator at 20℃ until analysis.

### 2.2 Extraction of FG

FG was extracted as previously described by Kaushik et al. (2017), with minor modifications, via hot water extraction and ethanol precipitation. Briefly, raw flaxseed (100 g) was soaked in distilled water for 2 hr at a flaxseed‐to‐water ratio of 1:15 (w/v), while stirring in a water bath at 80℃. The solution obtained was centrifuged at 10,000 × *g* for 10 min and the precipitate obtained by centrifugation was treated with three times the volume of 95% ethanol to precipitate the gum. The precipitate formed was collected via centrifugation at 10,000 × *g* for 10 min and dried in a blast drying oven at 50℃, followed by storage at 4℃ until further analysis. All the experiments were performed in triplicates.

### 2.3 Determination of FG yield

The following formula was used to calculate the yield (*Y*) of FG extracted from the different cultivars:

(1) $$Y\left(\%\right)=\frac{W_{g}}{W_{s}}\times 100$$

where *W*_g_ represents the weight of the FG powder (g) and *W*_s_ represents the weight of the raw flaxseed (g) used for FG extraction (Safdar et al., 2019).

### 2.4 Fourier‐transform infrared (FTIR) spectroscopy

FG sample particles (1–2 mg) were weighed, mixed with potassium bromide powder, pressed into tablets, and analyzed via FTIR spectroscopy (Nicolet is5, Thermo Fisher Scientific, Waltham, MA, USA). The resolution of the spectrum was 4/cm and the range was 4000–400/cm (Safdar et al., 2019). Scanning was performed 32 times.

### 2.5 X‐ray diffraction (XRD)

X‐ray diffractograms were obtained using a diffractometer (D8 Advance, Bruker, Billerica, MA, USA). The analysis was performed over a 2θ angle range of 5°–55°, and the sample was digitally scanned at a scan rate of 1°/min at increments of 0.05° (Kaushik et al., 2017).

### 2.6 Scanning electron microscopy (SEM)

To determine the surface attributes of the FG samples, the samples were characterized via SEM (GeminiSEM 500, ZEISS, Jena, Germany) as described by Hebeish et al. (2014), with slight modifications. Briefly, powdered FG samples were fixed to the surface of the carrier using conductive glue. Thereafter, the sample was immediately sputter‐coated with a gold target, and photographs were obtained under an accelerating voltage of 5 kV.

### 2.7 Measurement of zeta potential

Laser Doppler microelectrophoresis (Zetasizer Nano ZS‐90, Malvern Instruments, Malvern, UK) was performed to measure the zeta potential of FG solutions under different pH conditions. The FG powder was dissolved in distilled water to form a 0.1% (w/v) solution and then placed in the microelectrophoresis measurement chamber. To adjust the pH of the samples to 2.0–8.0, 0.1 M HCl or NaOH was used. All the measurements were performed in triplicates at 25℃, and the average value was determined (Kaushik et al., 2017).

### 2.8 Statistical analysis

The data obtained were expressed as the mean ±standard deviation of the triplicate tests. SPSS software v20.0 (IBM Inc., Chicago, IL, USA) was used for data analysis. Further, the Duncan test was used to analyze significant differences between the obtained experimental values. *p* < .05 was considered a statistically significant difference.

## 3 RESULTS AND DISCUSSION

### 3.1 FG yield

Hot‐water extraction is a traditional extraction method that does not require special conditions or equipment (Gu & Pan, 2014), and in this study, we examined the effect of the extraction conditions on the yield of FG. Significant differences among the different cultivars were observed with regard to this parameter (*p* < .05; Table 1). Further, the highest FG yield was obtained for FG1 (7.36%), whereas the lowest yield was obtained for FG4 (5.83%). In a previous study by Qian, the highest yield of FG was determined to be 9.7% at 25℃, based on extraction at a flaxseed‐to‐water ratio of 1:12 (Qian et al., 2012). Our yield of FG was lower than this previously reported value (Qian et al., 2012) but was 3.3% higher than that reported by Roulard et al., (2016). These differences in the FG yields of the different cultivars could be attributed to the differences between the environmental and climate characteristics corresponding to the different production areas, as well as differences in the degree of maturity of the flaxseed samples used. During processing, the extraction yield of FG is an important economic factor. Therefore, under the same extraction conditions, the cultivar exhibiting a higher yield will have a better economic value.

**TABLE 1:** Yield of FG extracted from different flaxseed cultivars

Cultivar	Yield (%)
FG1	7.36 ± 0.22^a^
FG2	6.12 ± 0.11^c^
FG3	7.18 ± 0.15^ab^
FG4	5.83 ± 0.31^c^
FG5	6.05 ± 0.25^c^
FG6	6.87 ± 0.27^b^

Note

Numbers in a row with different superscript are significantly different.

### 3.2 Fourier‐transform infrared spectroscopy

**FIGURE 1:**
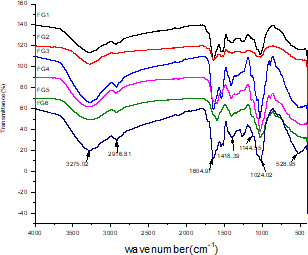
FTIR spectra of FG samples extracted from different flaxseed cultivars. Abbreviations: FG, flaxseed gum; FITR, Fourier‐transform infrared

The FTIR spectra of the FG samples (Figure 1) showed six characteristic bands with numerous peaks, ranging from 526.95 to 3,275.02/cm. The absorption peak at 3,275.02/cm was wide and could be attributed to the stretching vibration of non‐free hydroxyl (OH) groups (Li et al., 2018) forming hydrogen bonds with each other (Skwarek et al., 2017). The carbonyl (C = O) group asymmetric stretching vibration at 1604.97/cm resulted from the presence of uronic acid or bound water in FG. Further, the features extending around 1,418.39 and 1,604.97/cm could be attributed to the symmetric and asymmetric vibrations of the carboxylate (COO) group, respectively (Razmkhah et al., 2016) and the area between 800 and 1200/cm was considered as the carbohydrate fingerprint area, which can serve as an indicator of the differences between the gum structure (Sherahi et al., 2017). Further, the broad extension between 1,000 and 1,200/cm corresponded to a typical polysaccharide peak, while the peak at 1,144.55/cm could be attributed to the presence of glycosidic bonds, that is, C–OC–C and C–OH. Additionally, the absorption peak at 1,024.02 cm indicated the presence of dextran units or the CO stretching vibration in the pyranose ring. The absorption peak at 526.95/cm possibly resulted from the bending of the polymer backbone and could be attributed to the triple‐deteriorated bending mode of the vibration of the O–P–O bond in the phosphate group. Thus, the FTIR spectra of the FG samples showed typical polysaccharide absorption peaks that slightly differed from those reported previously owing to differences in the varieties examined as well as the differences in their monosaccharide contents.

### 3.3 XRD analysis

**FIGURE 2:**
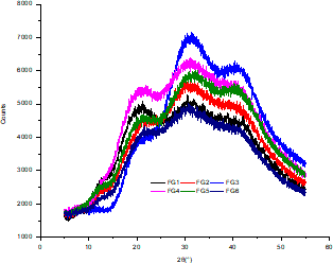
XRD patterns of FG samples extracted from different flaxseed cultivars. Abbreviations: FG, flaxseed gum; XRD, X‐ray diffractometry

XRD is primarily employed to study the grain size, orientation, and crystallinity of different materials. In a carbohydrate‐based linseed gum, a large number of OH groups in biopolymer molecules may lead to the formation of intramolecular and intermolecular hydrogen bonds, which can cause varying degrees of junction consistency and amorphous transformation. Additionally, sharp and broad peaks in X‐ray diffractograms are related to crystal characteristics and an amorphous structure, respectively. The X‐ray diffractograms of the FG samples, which are shown in Figure 2, exhibited broad peaks with 2θ values of approximately 21°, 30°, and 41°. Further, the XRD patterns of the FG samples showed a significant peak at 20° (2θ). This peak could be attributed to a covalent bond that is capable of promoting electrostatic interactions. The low intensity of this peak indicated that the structure of the extracted FG samples lacked crystallinity or an orderly arrangement. Consistent with our results, Devi & Bhatia (2019) also reported that flaxseed mucilage is a typical amorphous substance with an XRD pattern that shows no sharp peaks. Notably, sample FG3 showed a peak with greater intensity at 31°, indicating that it consisted of a small crystalline region that may be related to its source. Based on our results, we could conclude that owing to their different sources, most of the FG samples consisted of amorphous regions, while a few samples with amorphous regions consisted of a small crystalline area.

### 3.4 SEM analysis

**FIGURE 3:**
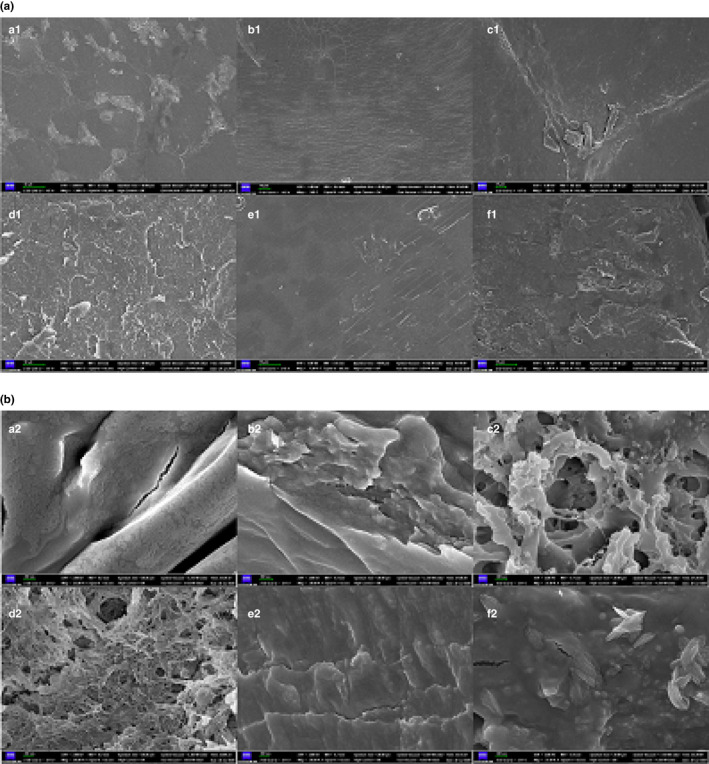
SEM images of FG samples extracted from different flaxseed cultivars: (a) at 1,000× magnification; and (b) at 20,000× magnification. Abbreviations: FG, flaxseed gum; SEM, scanning electron microscopy

We examined the surface morphology of the extracted FG samples via SEM. The micrographs of the different FG samples (Figure 3a) showed that all the FG powder particles (FG1–FG6) exhibited a smooth and irregular surface with a scaly structure. This was typically observed at a magnification of 1,000× in powders dried in hot air ovens. Similar results have been reported in previous studies (Kaushik et al., 2016; Wu et al., 2012). Further, the formation of a scaly FG structure during oven drying may be due to the rapid evaporation of the water molecules within the FG particles during the final drying stage, resulting in a rapid shrinkage of the particle surface. Furthermore, the microscopic view at a higher magnification of 20,000× (Figure 3b) showed a rough and flaky structure for FG1, FG2, and FG5, and a large reticulated void structure, fine honeycomb structure, and granular structure for FG3, FG4, and FG6, respectively.

### 3.5 Zeta potential

**FIGURE 4:**
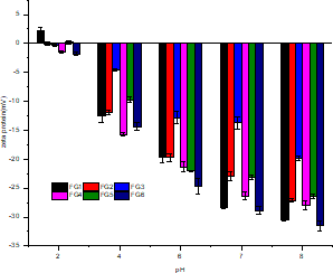
Zeta potential of FG samples extracted from different flaxseed cultivars. Abbreviation: FG, flaxseed gum

The variation of the zeta potentials of the FG samples in the pH range of 2.0–8.0 is shown in Figure 4 (*p* < .05). The maximum potential of the FG samples was observed at pH 8.0, and the potential was negative. Among all the FG samples, FG6 showed the maximum zeta potential (−31.5 mV), followed by FG1 (−30.4 mV), FG4 (−27.9 mV), FG2 (−27.1 mV|), FG5 (−26.4 mV), and FG3 (−19.8 mV). These different zeta potentials were consistent with their respective anionic characteristics. This observation could be primarily attributed to the decrease in the neutral monosaccharide to acidic monosaccharide ratio or the gradual increase in the acidic monosaccharide content of the FG samples, leading to changes in the observed charges. Additionally, it has been reported that the isoelectric point of flaxseed protein isolate is pH 4.2 (Kaushik et al., 2017). Owing of the low protein content of the FG samples extracted from the different flaxseed cultivars, the determination of the protein content of the samples was challenging; hence, no obvious changes in charge were observed close to the isoelectric point.

The intensity of the zeta potentials of the FG samples decreased with decreasing pH and reached zero at a pH of approximately 2.0. This observation is consistent with that reported by (Kaushik et al., 2017) When the pH was below 4.2, the protein was positively charged; however, as the protein content decreased, part of the positive charge was neutralized. Thus, there was no positive potential. However, when the pH was above 4.2 (exceeding the isoelectric point), the protein was negatively charged.

## 4 CONCLUSIONS

In this study, the diverse structural properties of FG samples obtained from six flaxseed cultivars were examined, and significant variations in the FG yields corresponding to these different cultivars (*p* < .05) were observed. However, all the samples showed typical polysaccharide absorption peaks, amorphous structures, and the presence of negatively charged macromolecules. Additionally, slight variations that could be attributed to the differences in the environmental and climate characteristics corresponding to their different production areas were noted. In conclusion, these diverse structural and functional properties of the FG samples extracted from different cultivars can serve as a theoretical basis for their application in food processing; this aspect should be further examined in future studies.

## CONFLICT OF INTEREST

There is no conflict of interest to declare.

## AUTHOR CONTRIBUTIONS

**Xuejiao Ren:** Conceptualization (lead); Data curation (lead); Funding acquisition (lead); Methodology (lead); Project administration (lead); Resources (lead); Validation (lead); Writing‐original draft (lead); Writing‐review & editing (lead). **Huili He:** Methodology (supporting); Validation (supporting). **Tuoping Li:** Conceptualization (equal); Project administration (equal); Writing‐review & editing (equal).

## ETHICAL APPROVAL

This research does not involve any studies with human and animal testing.

## Data Availability

All data included in this study are available upon request by contact with the corresponding author.
